# Intravenous administration of human mesenchymal stem cells derived from adipose tissue and umbilical cord improves neuropathic pain via suppression of neuronal damage and anti-inflammatory actions in rats

**DOI:** 10.1371/journal.pone.0262892

**Published:** 2022-02-14

**Authors:** Kanako Miyano, Minori Ikehata, Kaori Ohshima, Yuki Yoshida, Yasuhiro Nose, Sei-ichi Yoshihara, Katsuyuki Oki, Seiji Shiraishi, Miaki Uzu, Miki Nonaka, Yoshikazu Higami, Yasuhito Uezono

**Affiliations:** 1 Department of Pain Control Research, The Jikei University School of Medicine, Nishishimbashi, Minato-ku, Tokyo, Japan; 2 R&D Department, Biomimetics Sympathies Inc., Aomi, Koto-ku, Tokyo, Japan; 3 Pathology, Immunology and Microbiology, Graduate School of Medicine, The University of Tokyo, Hongo, Bunkyo-ku, Tokyo, Japan; 4 Laboratory of Molecular Pathology and Metabolic Disease, Faculty of Pharmaceutical Sciences, Tokyo University of Science, Yamazaki, Noda, Chiba, Japan; 5 Division of Cancer Pathophysiology, National Hospital Organization Kure Medical, Kure, Hiroshima, Japan; 6 Vitrigel Project, Institute of Agrobiological Sciences, National Agriculture and Food Research Organization, Tsukuba, Ibaraki, Japan; Hokkaido Daigaku, JAPAN

## Abstract

Mesenchymal stem cells (MSCs), which are isolated from adipose tissue (AD-MSCs), umbilical cord (UC-MSCs), or bone marrow, have therapeutic potential including anti-inflammatory and immunomodulatory activities. It was recently reported that MSCs are also effective as a therapeutic treatment for neuropathic pain, although the underlying mechanisms have yet to be resolved. Therefore, in this study, we investigated the effects of human AD- and UC-MSCs on neuropathic pain and its mechanisms using rat models of partial sciatic nerve ligation (PSNL). AD- or UC-MSCs were intravenously administered 4 days after PSNL. Antinociceptive effects were then evaluated using the von Frey and weight-bearing tests. We found that, 3–9 days after the administration of AD- or UC-MSCs to PSNL-exposed rats, both the mechanical threshold and differences in weight-bearing of the right and left hind paws were significantly improved. To reveal the potential underlying antinociceptive mechanisms of MSCs, the levels of activation transcription factor 3- and ionized calcium-binding adapter molecule 1-positive cells were measured by immunohistochemical analysis. AD- and UC-MSCs significantly decreased the levels of these proteins that were induced by PSNL in the dorsal root ganglia. Additionally, UC-MSC significantly improved the PSNL-induced decrease in the myelin basic protein level in the sciatic nerve, indicating that UC-MSC reversed demyelination of the sciatic nerve produced by PSNL. These data suggest that AD- and UC-MSCs may help in the recovery of neuropathic pain via the different regulation; AD-MSCs exhibited their effects via suppressed neuronal damage and anti-inflammatory actions, while UC-MSCs exhibited their effects via suppressed neuronal damage, anti-inflammatory actions and remyelination.

## Introduction

Neuropathic pain is a chronic pain condition that can be initiated by injury or perturbation of either the peripheral or central nervous system [1]. Current therapies for neuropathic pain are limited, and analgesic drugs, such as nonsteroidal anti-inflammatory drugs and opioids, are often ineffective [1]. Therefore, the development of effective analgesics for the management of neuropathic pain represents an important unmet medical need [1].

Mesenchymal stem cells (MSCs) isolated from adipose tissue, umbilical cord, bone marrow, dental pulp, or amnion membrane have the ability to self-replicate and self-propagate in addition to being pluripotent [2]. Importantly, MSCs do not express the major histocompatibility complex class II cell surface receptor HLA-DR, indicating high immunological tolerance [3–5]. Therapeutic approaches using MSCs can be divided into two strategies: cell replacement therapy, which makes use of the multi-differentiation ability of MSCs thus repairing the damaged tissue, and cell humoral factor therapy, which uses the actions of several factors released from MSCs, including their immunoregulatory, anti-inflammatory, and angiogenic actions [2, 3, 5, 6]. Therefore, treatments using MSCs represent a novel therapeutic approach that produces multi-pharmacological actions.

Previous animal and clinical reports have revealed that MSCs can exert a wide range of therapeutic responses, including anti-inflammatory, immunomodulatory, neuroprotective, and anti-fibrotic effects in addition to promoting angiogenesis [2, 3, 5]. The first Food and Drug Administration-approved therapeutic product using MSCs was for the treatment of acute graft-versus-host disease. Excitingly, previous animal studies using neuropathic pain models have demonstrated that MSCs obtained from rodents or human produce analgesic effects through anti-inflammatory actions [7–11]. However, few studies have analyzed the actions of MSCs on neuronal damage, such as demyeliation in neuropathic pain models.

In the present study, we focused on MSCs, which could be abundantly obtained from human adipose (AD-MSC) and umbilical cord (UC-MSC) tissues with low invasiveness to develop a clinical application. The aim of this study was to evaluate the therapeutic effects of intravenous administration of human MSCs using rat models of partial sciatic nerve ligation (PSNL)-induced pain, which is one of the surgically injured nerve models. We revealed that both AD- and UC-MSCs significantly suppressed PSNL-induced pain and investigated the potential mechanisms underlying the antinociceptive actions of intravenously administered MSCs.

## Materials and methods

### Experimental animals for the PSNL model

Male Sprague Dawley (SD) rats aged 4 weeks (Clea-Japan, Tokyo, Japan) were housed individually with a 12-h light-dark cycle (lights on at 08:00 AM) at constant temperature and humidity, with *ad libitum* access to food and water. The rats were acclimated to laboratory conditions for 1 week prior to the experiment. All experiments were conducted in accordance with the ethical guidelines of the International Association for the Study of Pain and approved by the Committee for Ethics of Animal Experimentation of National Cancer Center (Approval No. T17-004). During the experiments, efforts were made to minimize the suffering of the animals as well as the number of animals used.

### The PSNL model

SD rats (5 weeks-old male) were anesthetized with isoflurane, and partial ligation of the sciatic nerve was performed by tying off the distal one-third to one-half of the sciatic nerve according to the procedure described by Seltzer et al. [12]. In rats that underwent sham surgery, the nerve was exposed using the same procedure, but was not ligated.

### Cell culture

AD- and UC-MSCs were provided from Biomimetics Sympathies Inc. The stromal vascular fraction (SVF) was isolated from adipose and umbilical cord tissues by a combination of collagenase enzymatic digestion (900 U/mL, 1 h at 37°C) with a rotary shaker and centrifugation (1,000 × *g*, 5 min) as previously described [13]. The isolated SVF was suspended in phosphate-buffered saline (PBS), passed through a 70-μm filter, and centrifuged at 400 × *g* for 5 min to obtain the SVF. The collected SVF was seeded into flasks (CellBIND^®^ T-Flask; Corning Inc., NY, USA) in animal origin-free medium (MS-E0001, BioMimetics Sympathies Inc., Tokyo, Japan) to expand AD-MSCs and UC-MSCs, which was biologically safe because it was free from animal- and human-derived materials in addition to being serum-free (culture conditions: 5% CO_2_, 37°C). Medium exchange was performed at 2-day intervals. After reaching semi-confluence, subculture was performed using TrypLE^™^ Select (Thermo Fisher Scientific Inc., MA, USA). Surface antigen marker properties of the AD- and UC-MSCs used in this study were investigated using several antibodies and detected using Cytomics FC500 flow cytometer (Beckman Coulter Inc., CA, USA). The percentages of antigen marker-positive cells were as follows: AD-MSC: CD31, 0.7%; CD45, 0.2%; CD73, 97.8%; CD90, 98.2% and UC-MSC: CD31, 0.2%; CD45, 0.1%; CD73, 99.7%; CD90, 99.9%, indicating that these MSCs showed typical CD antigen expression as described by Dominici et al. [4]. Adult human adipose and umbilical cord tissues were collected from volunteers undergoing orthopedic surgery following the ethical guidelines of the Sun Field Clinic in Tokyo, Japan and the Narita Ladies Clinic in Saitama, Japan, respectively. In all studies using human materials, informed consent was obtained from the volunteers before the surgical procedure.

### MSC administration

After reaching confluence, adherent cells were trypsinized using TrypLE Select (Thermo Fisher Scientific Inc., MA, USA). After centrifugation, the cells were immediately washed, counted, and suspended in PBS (Sigma-Aldrich, St. Louis, MO, USA). Four days after ligation of the sciatic nerves in SD rats, 1 mL of MSCs (5 × 10^6^ cells/mL in PBS) were intravenously injected.

### Pain assessment

To evaluate pain behaviors, the von Frey and dynamic weight bearing (DWB) tests were performed. The von Frey test was performed to assess mechanical threshold, as previously described [14]. According to the procedure by Chaplan et al. [15], the 50% paw withdrawal threshold was measured using the up-down method with a von Frey filament (0.01–15.0 g). The DWB test (Bioseb, France) is a test for assessing spontaneous pain in freely moving rodents, which is based on an instrumented-floor cage and a combined video acquisition system. In this study, data are shown as the weight ratio of the left/right hind paw (ligation side/non-ligation side), according to the procedure by Quadros et al. [16].

### Histological analysis

The sciatic nerves (a length of approximately 5 mm each on the spinal and terminal side from the ligation site) and L4 and L5 dorsal root ganglia (DRG) of rats on days 4–11 after PSNL (days 3–7 after intravenous injection of MSCs) were rapidly dissected and fixed with 4% paraformaldehyde prior to being embedded in paraffin. The sections were cut at a thickness of 8 μm and blocked using Blocking One Histo (Nacalai Tesque, INC., Kyoto, Japan) for 1 h at 25 ± 2 °C. Each primary antibody was diluted in PBS containing Blocking One (Nacalai Tesque, 1:20) and the sections were incubated for 1 h at 25 ± 2 °C. The following antibodies were used: rabbit activation transcription factor 3 (ATF-3, 1:100; Santa Cruz, Dallas, TX, USA), goat ionized calcium-binding adapter molecule 1 (Iba-1 1:250; FUJIFILM Wako Pure Chemical Corporation, Osaka, Japan), rabbit myelin basic protein (MBP, 1:500; Abcam), mouse neuronal specific nuclear protein (NeuN, 1:200; Abcam), and chicken neurofilament heavy polypeptide (NF200, 1:250; Abcam). The sections were rinsed and incubated with secondary goat polyclonal anti-rabbit antibody conjugated with Alexa Fluor^®^ 594 (1:1000; Abcam) or chicken anti-rabbit antibody conjugated with Alexa Fluor^®^ 594 (1:1000; Molecular Probes, Eugene, OR, USA), and goat polyclonal anti-mouse or chicken Alexa Fluor^®^ 488 (1:1000; Abcam) for 1 h at 25 ± 2 °C. The slides were then cover-slipped with PermaFluor Aqueous mounting medium (Thermo Fisher Scientific, Waltham, MA, USA) with 1 μg/mL of DAPI (Bio-Rad Laboratories, Inc., Hercules, CA, USA), and the immunofluorescent labeling was captured using a fluorescence microscope (BZ-X710; Keyence, Osaka, Japan). In the present study, 1–2 sections/ animal were stained and images (1–2 images/ sections, ×20; 550×700 μm, ×40; 275×350 μm) were observed. ATF-3- and NeuN-positive cells were quantified using the following formula: positive cells/area using ImageJ software (National Institutes of Health, Bethesda, MD, USA). The total fluorescence intensity/ area for Iba-1, MBP and NF200 was calculated using ImageJ software.

### Statistical analysis

All data are presented as mean ± standard error of the mean. Statistical analyses were performed using one- or two-way analysis of variance followed by Bonferroni’s multiple comparisons test (GraphPad Prism 6, GraphPad Software, San Diego, CA, USA). A probability value (*p*) < 0.05 was considered statistically significant.

## Results

### Intravenous injection of AD- and UC-MSCs significantly improved the mechanical pain threshold and weight distribution of the hind paws

**Fig 1:**
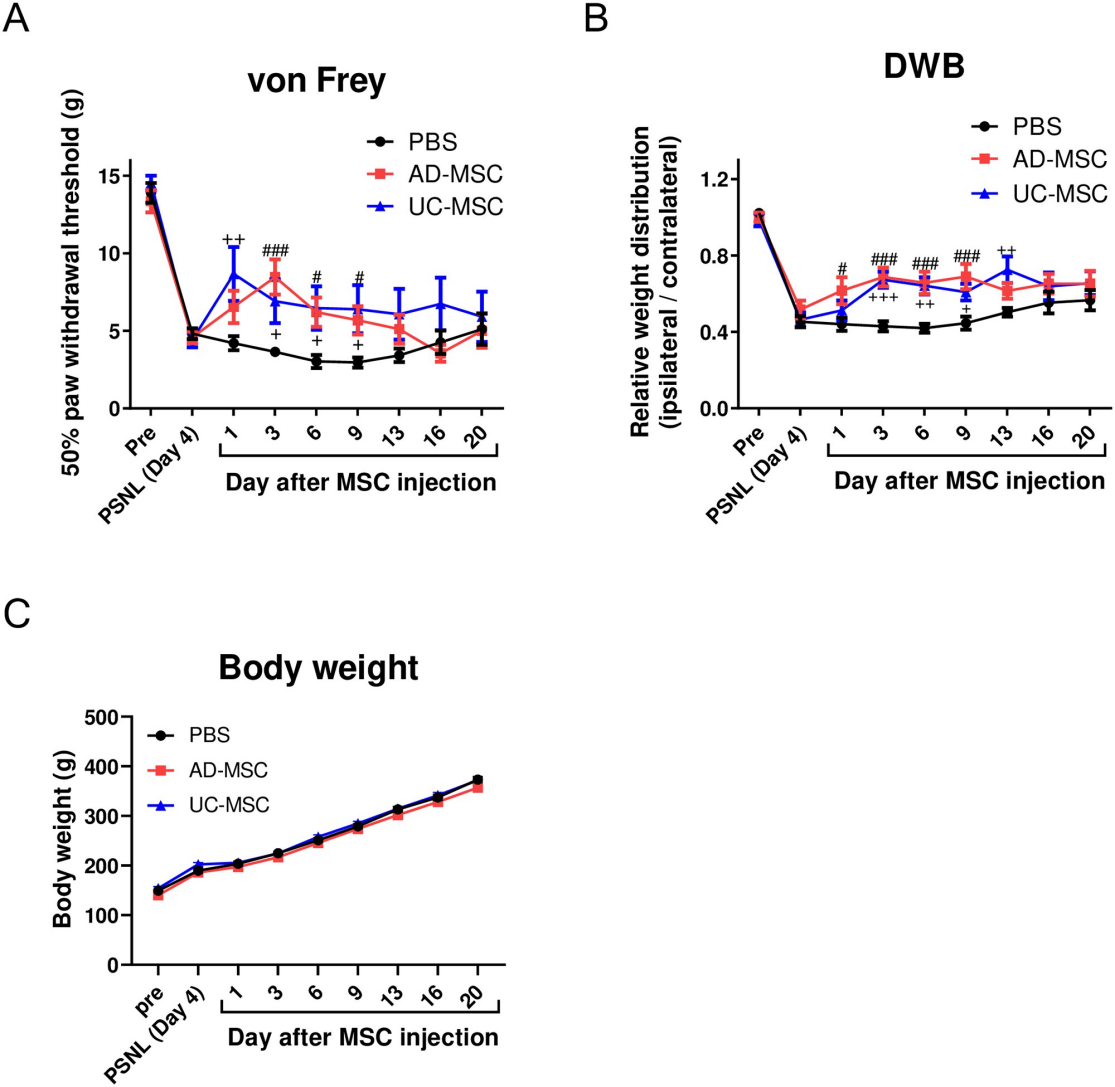
Effects of AD- and UC-MSCs on PSNL-induced pain. On day 4 after PSNL, AD-MSCs, UC-MSCs (5 × 10^6^ cells/mL), or its vehicle (PBS; 1 mL) was administered intravenously to rats, and the von Frey (A, PBS; n = 17, AD-MSC; n = 18, UC-MSC; n = 10) and DWB tests (B, PBS; n = 18, AD-MSC; n = 16, UC-MSC; n = 12) were performed, and body weight (C, PBS; n = 18, AD-MSC; n = 16, UC-MSC; n = 12) was measured. The data are expressed as mean ± standard error of the mean. ^#^ and ^###^ indicate *p* < 0.05 and 0.001, respectively, sham vs. AD-MSC group; ^+^, ^++^, and ^+++^ indicate *p* < 0.05, 0.01, and 0.001, respectively, sham vs. UC-MSC group: Bonferroni’s multiple comparison test was performed following a two-way analysis of variance.

We examined the effects of MSCs on neuropathic pain using the von Frey and DWB tests. Compared with sham treatment, the mechanical pain threshold significantly decreased from day 4 to day 24 (S1 Fig). The weight distribution of hind paws also significantly decreased from day 4 to day 24 (S1 Fig). On the other hand, there was no difference in body weight between the sham and PSNL rats before and after PSNL (S1 Fig). Next, we investigated the effects of AD- and UC-MSCs on PSNL-induced pain. On day 4 after PSNL, AD-MSCs, UC-MSCs (5 × 10^6^ cells/mL, or the vehicle treatment (PBS; 1 mL) was administered intravenously to rats. In the von Frey test, administration of AD-MSCs significantly improved the PSNL-induced decrease in mechanical pain threshold on days 3–9 post-injection, while administration of UC-MSCs significantly ameliorated this decrease on days 1–9 post-injection (Fig 1A). In the DWB test, administration of AD-MSCs ameliorated the PSNL-induced decrease in weight distribution of the hind paws on days 1–9 post injection, while administration of UC-MSCs significantly improved weight distribution of the hind paws on days 3–13 post-injection (Fig 1B). Importantly, body weight was not significantly different between the MSCs-treated rats and controls (Fig 1C). Based on these findings, we focused on days 3–7 post intravenous injection of MSCs to investigate the underlying mechanisms of the observed MCSs-induced analgesic actions using rat models of PSNL.

### AD- and UC-MSCs significantly suppresses the increase in ATF-3-positive neurons induced by PSNL in DRG

**Fig 2:**
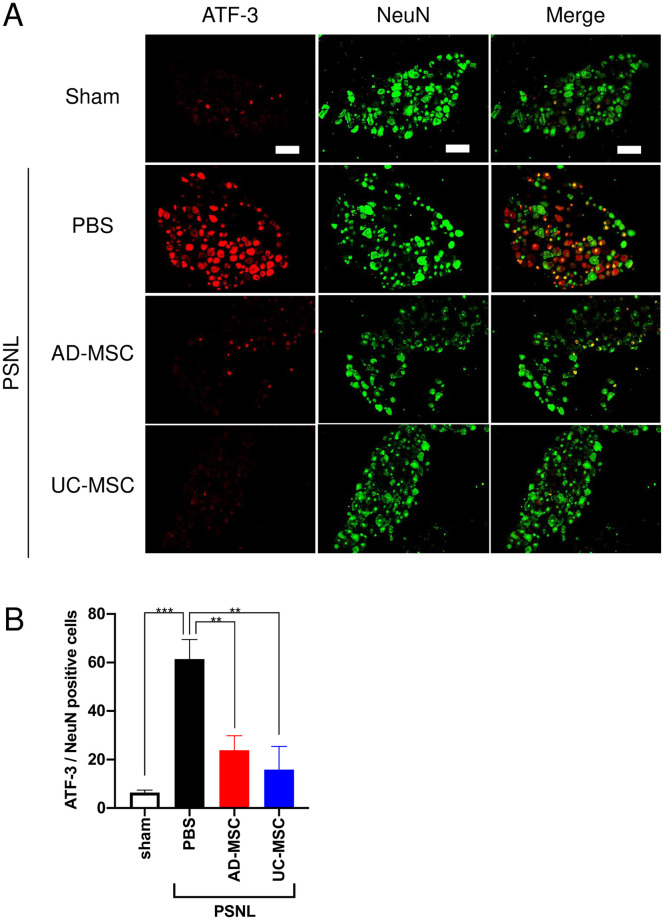
Changes in expression of ATF-3 and NeuN in the DRGs of PSNL-exposed rats and the effects of treatment with MSCs. On day 4 after partial sciatic nerve ligation (PSNL), AD-MSCs, UC-MSCs (5 × 10^6^ cells/mL), or its vehicle (PBS) was administered intravenously. On day 3 after injections, DRG were dissected from rats. The protein expression of MBP and NF200 in the DRG was measured by immunohistochemical analysis (A). The data are expressed as mean ± standard error of the mean (B, sham; n = 5, PBS; n = 6, AD-MSC; n = 4, UC-MSC; n = 4). ** and *** indicate p < 0.01 and p < 0.001, respectively: Bonferroni’s multiple comparison test was performed following a one-way analysis of variance. Scale bar = 100 μm.

After neuronal damage or stress, ATF-3, a neuronal injury marker, is upregulated on DRG neurons [17]. To investigate the expression levels of ATF-3 in the DRG using the PSNL model, double-immunostaining with anti-ATF-3 and anti-NeuN antibodies, one of neuronal marker, was performed. Compared to the sham group, there was a significant increase in ATF-3-positive cells in the DRG of PSNL-exposed rats (PBS group), which were co-expressed with NeuN (Fig 2A). The quantification results indicated that 61.4 ± 4.6% of NeuN-positive cells was co-expressed with ATF-3 (Fig 2B). Injection of AD- and UC-MSCs significantly suppressed the PSNL-induced increase in ATF-3-positive cells (Fig 2B).

### AD- and UC-MSCs significantly suppress the PSNL-induced accumulation of macrophages in the dorsal root ganglia

**Fig 3:**
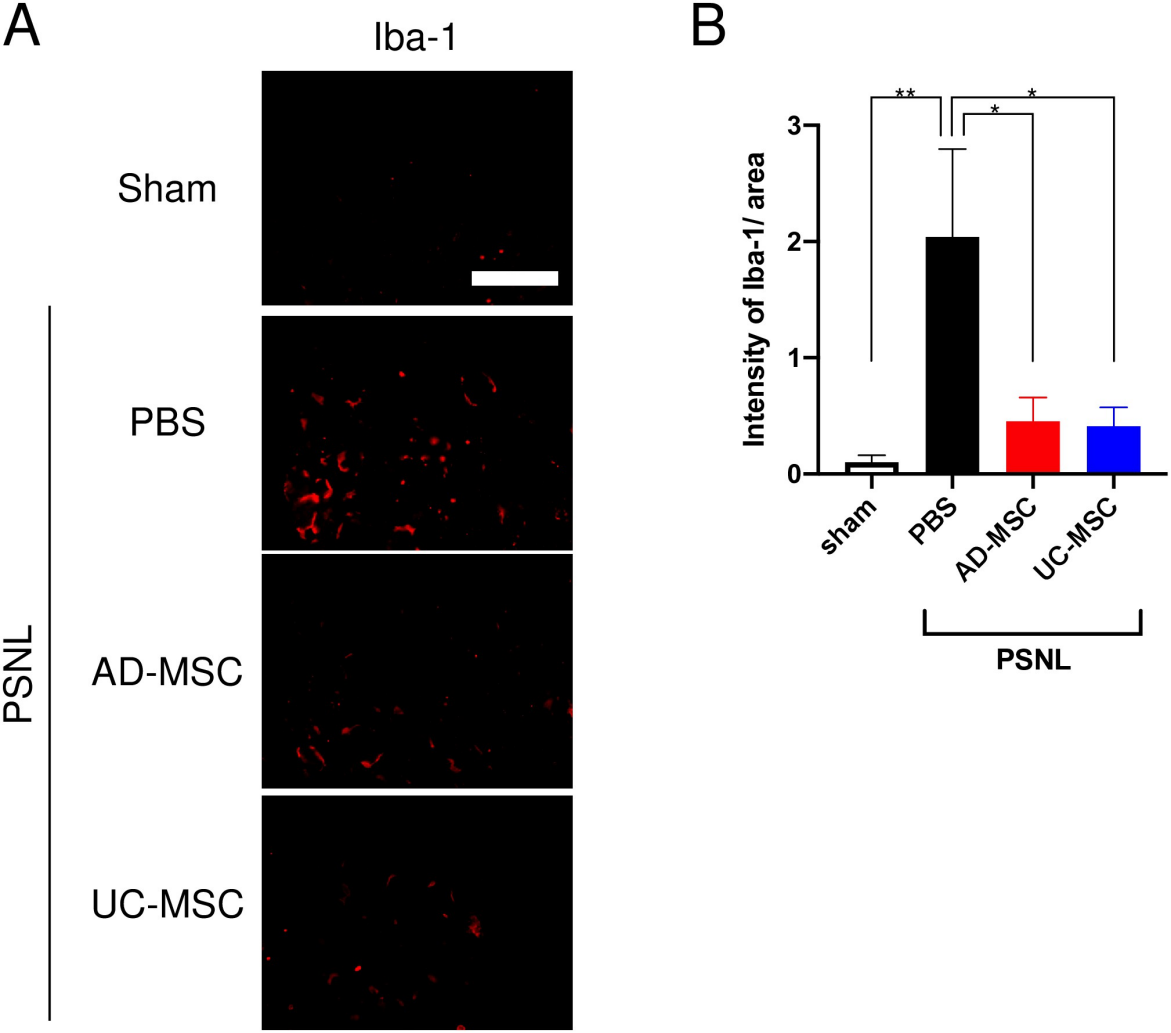
Change in the number of Iba-1-positive cells in the dorsal root ganglia (DRG) of PSNL-exposed rats and the effect of treatment with MSCs. On day 4 after partial sciatic nerve ligation (PSNL), AD-MSCs, UC-MSCs (5 × 10^6^ cells/mL), or its vehicle (PBS) was administered intravenously. On day 3 after injections, DRG were dissected from the rats. The protein expression of Iba-1 in the DRG was measured by immunohistochemical analysis (A). The data are expressed as mean ± standard error of the mean (B, sham; n = 4, PBS; n = 3, AD-MSC; n = 3, UC-MSC; n = 4). * and ** indicate p < 0.05 and 0.01, respectively: Bonferroni’s multiple comparison test was performed following a one-way analysis of variance. Scale bar = 100 μm.

Many studies have demonstrated that the accumulation of macrophages plays an important role in neuropathic pain [18–22]. Therefore, to reveal the mechanism(s) underlying the MSC therapeutic effect on neuropathic pain, we analyzed changes in Iba-1-positive cells, a marker of macrophages, in DRG using immunohistochemical analysis. Compared to the sham group, Iba-1-positive cells increased in the DRG of PSNL-exposed rats (PBS groups, Fig 3A). Conversely, administration of AD- and UC-MSCs suppressed the PSNL-induced accumulation of Iba-1-positive cells in DRG (Fig 3A). As shown in Fig 3B, exposure to PSNL significantly induced an increase in Iba-1-positive cells, which was significantly decreased by treatment with AD- and UC-MSCs.

### UC-MSCs improved demyelination of the sciatic nerves in the PSNL model

Peripheral nerve injury-induced demyelination is correlated with neuropathic pain [21, 23]. In the sciatic nerves of the sham group at days 4, 7, and 11, Luxol Fast Blue-positive cells were approximately uniform in size and arranged with regularity (S2 Fig). Conversely, in the sciatic nerves of the PSNL group at days 4, 7, and 11, Luxol Fast Blue-positive cells were different in size, and there were a few strongly positive cells, indicating myelin denaturation from day 4 to day 11.

**Fig 4:**
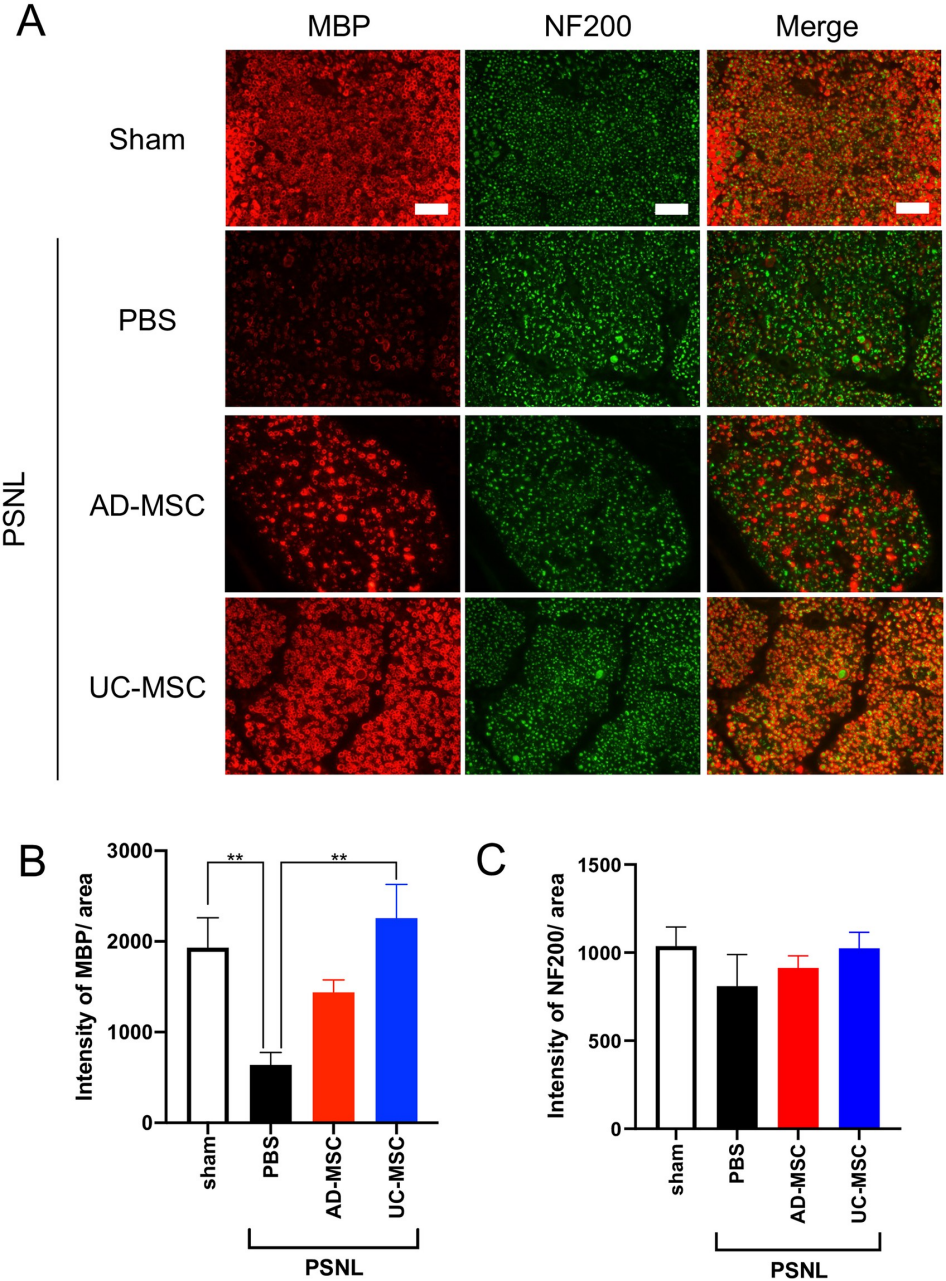
Changes in expression of myelin basic protein (MBP) and neurofilament heavy polypeptide (NF200) in the sciatic nerves of PSNL-exposed rats and the effects of treatment with MSCs. On day 4 after partial sciatic nerve ligation (PSNL), AD-MSCs, UC-MSCs (5 × 10^6^ cells/mL), or its vehicle (PBS) was administered intravenously. On day 3 after injections, sciatic nerves were dissected from rats. The protein expression of MBP and NF200 in the sciatic nerves was measured by immunohistochemical analysis (A). The data are expressed as mean ± standard error of the mean (B and C, sham; n = 4, PBS; n = 5, AD-MSC; n = 6, UC-MSC; n = 6). ** indicates p < 0.01: Bonferroni’s multiple comparison test was performed following a one-way analysis of variance. Scale bar = 50 μm.

Moreover, we analyzed changes in MBP expression in the sciatic nerves. As shown in Fig 4A, MBP expressed around NF-200-positive cells, a marker for A-fiber (myelinated nerve). Compared to the sham group, the PSNL group (PBS group) had significantly less MBP-positive cells in the sciatic nerves on day 7 (Fig 4A). The quantification results also indicated that PSNL induced a significant decrease in expression of MBP (Fig 4B). Treatment with AD-MSC showed a slight improvement but its changes were not significant. On the other hand, UC-MSC almost completely recovered the PSNL-induced decrease in expression of MBP, suggesting that UC-MSC reversed demyelination on days 7–11 after PSNL (day 3–7 after MSCs administration) (Fig 4B). There was no significant change in NF-200 positive cells (Fig 4C).

## Discussion

Our present study revealed that MSCs from either AD or UC significantly suppress PSNL-induced neuropathic pain. We also found that a single intravenous administration of AD- or UC-MSCs is sufficient to decrease PSNL-induced neuropathic pain, and that these antinociceptive effects last for at least 7 days (Fig 1). Analysis of the mechanisms underlying the analgesic properties of AD- and UC-MSCs revealed that, in DRG, these MSCs significantly suppressed the PSNL-induced accumulation of ATF-3- and Iba-1-positive cells (Figs 2 and 3). Moreover, in the sciatic nerves, UC-MSC improved the PSNL-induced decrease in MBP, but not AD-MSC (Fig 4). These data suggest that AD- and UC-MSCs may ameliorate neuropathic pain via the regulation of inflammation and reverse the demyelination of the sciatic nerves induced by PSNL.

In the present study, we demonstrated that a single intravenous administration of AD- or UC-MSCs produced similar antinociceptive actions. In addition to these MSCs, bone marrow-derived MSCs have also been reported to exhibit antinociceptive effects, which appeared within 24 h and were maintained for 5 days after administration [7], indicating that MSCs, regardless of their origin, reduce neuropathic pain for an extended time. In addition, most previous studies have shown that not only rodent- but also human-derived MSCs were administered to mice or rats, and observed a significant suppression of neuropathic pain with long-lasting analgesic effects via anti-inflammatory effects, such as decrease of interleukin-1β without rejection [8–11, 21]. These data suggest that actions of human and rodent MSCs on model animals of neuropathic pain using rodents might not so different, although further investigations are needed to carefully reveal the species difference between human and rodent MSCs.

It is important to analyze whether AD- and UC-MSCs are present in the affected area, such as DRG or sciatic nerve, in order to elucidate its analgesic mechanism. Liu et al. [24] reported that both intravenous and intrathecal transplantation of MSCs significantly attenuated neuropathic pain, and MSCs were found on the surface of the spinal cord and DRG. In addition, conditioned medium of MSCs derived from the bone marrow had antinociceptive effects that were maintained for 5 days [7]. Intrathecal injection of exosomes derived from human UC-MSCs have also been reported to reverse nerve ligation-induced mechanical and thermal hypersensitivities in rats at the initial and well-developed pain stages [25]. Based on these reports, the hypotheses for the antinociceptive mechanisms of MSCs made in the present study can be summarized as follows: 1) MSCs directly act on the DRG or sciatic nerves to produce anti-inflammatory and remyelination effects and 2) paracrine factors released from MSCs act on the DRG or sciatic nerves. We are currently investigating whether MSCs are present in the DRG and sciatic nerve.

Neuroinflammation and neuroimmune interactions contribute to the development of neuropathic pain through regulation of the functions of macrophages [19, 26]. Macrophages have been reported to respond to peripheral nerve injury [27]. Macrophages infiltrate DRG in nerve-injured animals, and activated macrophages produce pro-inflammatory cytokines, which can generate and maintain neuropathic pain [18–22, 26]. In the present study, PSNL induced an increase in both ATF-3- and Iba-1-positive cells in the DRG, suggesting that macrophages infiltrate the DRG by PSNL-induced neuronal damage. Numerous studies support the notion that cytokines, that are released mainly from macrophages and DRG neurons, lead to the development of neuropathic pain [18, 19, 22, 26, 28–32]. Our present study showed that treatment with AD- or UC-MSCs significantly reversed neuronal damage and the accumulation of macrophages. Shiue et al. reported that exosomes derived from MSCs suppressed nerve ligation-induced upregulation of Iba-1 [25]. Other studies have also shown that MSCs and conditioned medium derived from MSCs significantly decreased the expression levels of cytokines *in vivo* [7–11]. However, it is unclear how MSCs improve neuronal damage and the accumulation of macrophages in the DRG after nerve injury. Further studies are needed to reveal the mechanisms that mediate the actions of MSCs on the damaged neurons and macrophages.

To the best of our knowledge, this study is the first to reveal that UC-MSCs reduces the demyelination of the sciatic nerves using rat models of PSNL. In diabetic peripheral neuropathy models, bone marrow-derived MSCs have been shown to reduce myelin irregularities [23]. Furthermore, Wang et al. reported that exosomes derived from bone marrow-MSCs significantly reverse spinal cord injury-induced decreases in MBP [33]. In addition, Chu et al. demonstrated that cytokine-mediated NFκB activation was related to Schwann cell demyelination using a CCI model of neuropathic pain [34]. Moreover, tonsil-derived MSCs have been shown to differentiate into a Schwann cell phenotype and promote peripheral nerve regeneration [35]. Clinical studies also have shown that MSCs derived from bone marrow or UC improved multiple sclerosis, which results from demyelination by autoantibodies that bind to self-myelin antigens [36–38]. In the present study, AD-MSCs showed a slight improvement in demyelination, but the changes were not significant, despite a significant decrease in both the accumulation of macrophages and ATF-3-positive neurons, similar to UC-MSCs. These data suggest that actions other than suppression of neuronal damage and anti-inflammatory effects play key roles in UC-MSC-induced remyelination. Further investigations are needed to reveal the mechanisms that mediate UC-MSC-induced remyelination of the sciatic nerves.

For application to clinical practice, we examined the intravenous injection of AD- and UC-MSCs. Intravenous injection is one of the representative methods to administer drugs. AD- and UC-MSCs can be obtained in abundance with low invasiveness. AD-MSCs are considered to be autologous MSC therapy, while UC-MSCs are allogeneic MSC therapy. Many clinical studies have indicated that therapies with AD- and UC-MSCs are safe for humans [36–38]. Our present study using PSNL model rats showed that there was a slight difference between AC- and UC-MSCs, even though these MSCs had analgesic actions; analgesic actions induced by UC-MSC were via both anti-inflammatory actions and remyelination, while those by AD-MSC were mainly through anti-inflammatory actions. These data suggest that both MSCs might be a useful therapy for neuropathic pain, although further clinical studies are needed to investigate this treatment modality.

In conclusion, the present study revealed that a single intravenous administration of human AD- or UC-MSCs decreased PSNL-induced neuropathic pain; these analgesic effects lasted for at least 7 days and were associated with dual-pharmacological actions (anti-inflammatory actions and remyelination). Since there are very few effective treatments that can improve demyelination in clinical cases, AD- and UC-MSCs may be used not only for symptomatic therapy, but also as a novel therapy for neuropathic pain. This study provides further evidence supporting the use of MSCs in patients with neuropathic pain.

## Supporting information

S1 Fig Pain measurements in PSNL-exposed rats. In rats that received sham or partial sciatic nerve ligation (PSNL), the von Frey test (A, sham; n = 13, PSNL; n = 14) and dynamic weight bearing test (B, sham; n = 13, PSNL; n = 14) were performed, and body weight (C, sham; n = 13, PSNL; n = 14) was measured. The data are expressed as the mean ± standard error of the mean. ** and *** indicate p < 0.01 and p < 0.001 respectively, compared to the sham group: Bonferroni’s multiple comparison test was performed following a two-way analysis of variance.
(DOCX)

S2 Fig Luxol Fast Blue staining of the sciatic nerves in PSNL-exposed rats. On days 4, 7, and 11 after sham or partial sciatic nerve ligation (PSNL), the sciatic nerves were dissected from rats (n = 3 at each time point). The sciatic nerves were stained with Luxol Fast Blue to observe myelin using immunohistochemical analysis. Representative pictures of the sciatic nerves from sham or PSNL rats are shown. Arrows denote strongly stained Luxol Fast Blue-positive cells, which indicates myelin denaturation. Bar indicates 50 μm. This experiment was independently performed at least three times.
(DOCX)
