# Supplementary material for: Intravenous administration of human mesenchymal stem cells derived from adipose tissue and umbilical cord improves neuropathic pain via suppression of neuronal damage and anti-inflammatory actions in rats
Source: PLoS One. 2022 Feb 14;17(2):e0262892. doi: 10.1371/journal.pone.0262892 (PMC8843230; doi:10.1371/journal.pone.0262892)

**S1 Fig. Pain measurements in PSNL-exposed rats.** In rats that received sham or partial sciatic nerve ligation (PSNL), the von Frey test (A, sham; n = 13, PSNL; n = 14) and dynamic weight bearing test (B, sham; n = 13, PSNL; n = 14) were performed, and body weight (C, sham; n = 13, PSNL; n = 14) was measured. The data are expressed as the mean ± standard error of the mean. ** and *** indicate p < 0.01 and p < 0.001 respectively, compared to the sham group: Bonferroni’s multiple comparison test was performed following a two-way analysis of variance.

S1 Fig


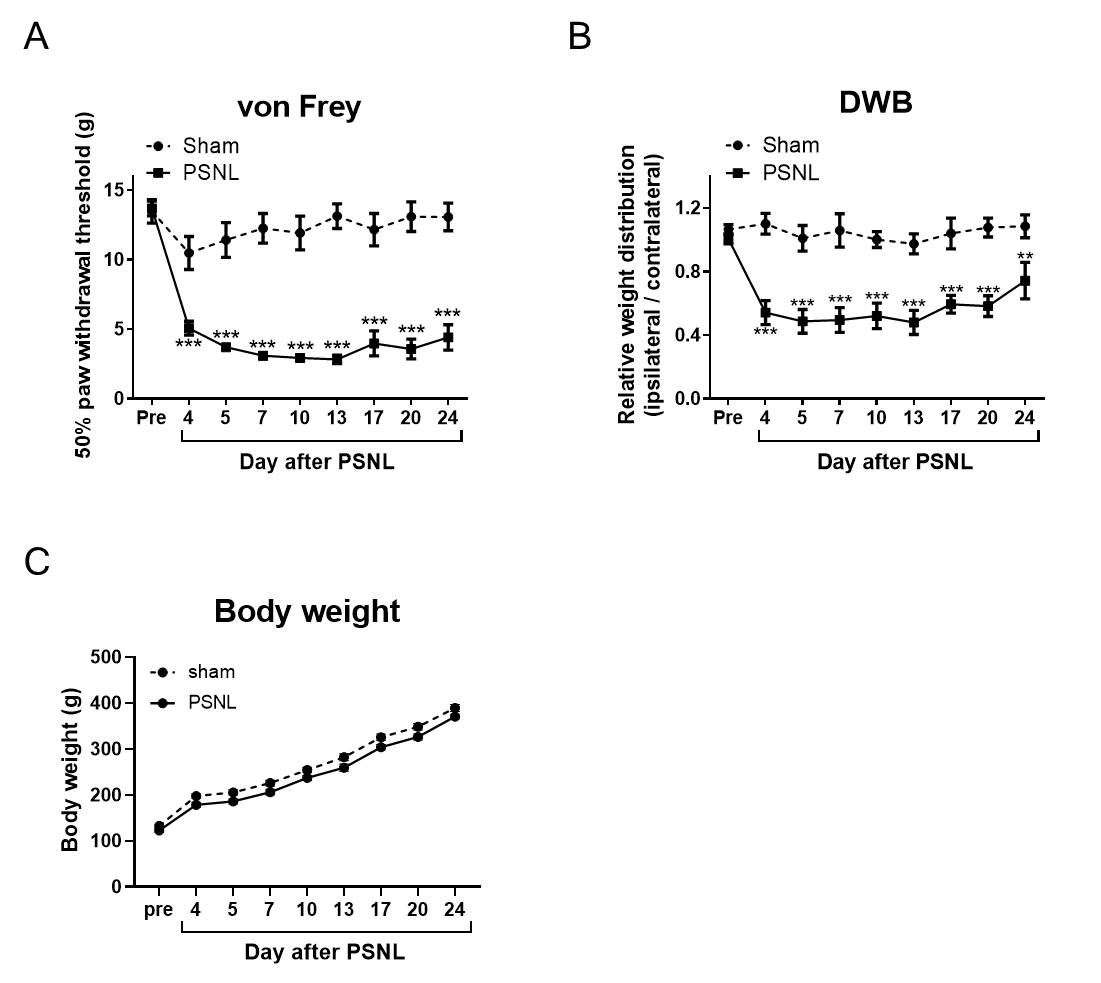

Supplement: S1 Fig — In rats that received sham or partial sciatic nerve ligation (PSNL), the von Frey test (A, sham; n = 13, PSNL; n = 14) and dynamic weight bearing test (B, sham; n = 13, PSNL; n = 14) were performed, and body weight (C, sham; n = 13, PSNL; n = 14) was measured. The data are expressed as the mean ± standard error of the mean. ** and *** indicate p < 0.01 and p < 0.001 respectively, compared to the sham group: Bonferroni’s multiple comparison test was performed following a two-way analysis of variance. (DOCX) [file pone.0262892.s001.docx]
