# Supplementary material for: Intravenous administration of human mesenchymal stem cells derived from adipose tissue and umbilical cord improves neuropathic pain via suppression of neuronal damage and anti-inflammatory actions in rats
Source: PLoS One. 2022 Feb 14;17(2):e0262892. doi: 10.1371/journal.pone.0262892 (PMC8843230; doi:10.1371/journal.pone.0262892)

**S2 Fig. Luxol Fast Blue staining of the sciatic nerves in PSNL-exposed rats.** On days 4, 7, and 11 after sham or partial sciatic nerve ligation (PSNL), the sciatic nerves were dissected from rats (n = 3 at each time point). The sciatic nerves were stained with Luxol Fast Blue to observe myelin using immunohistochemical analysis. Representative pictures of the sciatic nerves from sham or PSNL rats are shown. Arrows denote strongly stained Luxol Fast Blue-positive cells, which indicates myelin denaturation. Bar indicates 50 µm. This experiment was independently performed at least three times.

S2 Fig


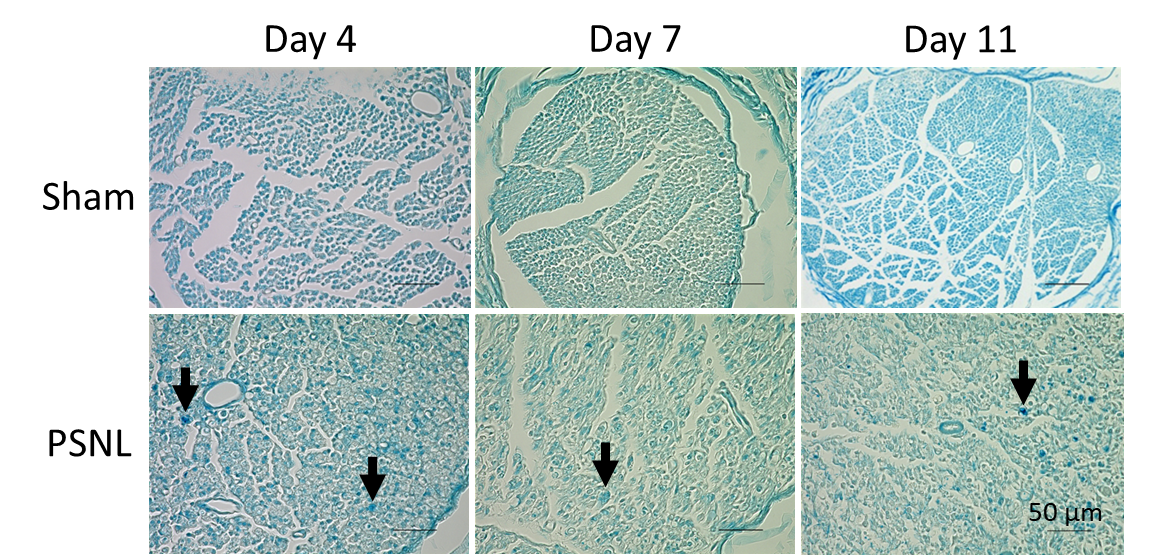

Supplement: S2 Fig — On days 4, 7, and 11 after sham or partial sciatic nerve ligation (PSNL), the sciatic nerves were dissected from rats (n = 3 at each time point). The sciatic nerves were stained with Luxol Fast Blue to observe myelin using immunohistochemical analysis. Representative pictures of the sciatic nerves from sham or PSNL rats are shown. Arrows denote strongly stained Luxol Fast Blue-positive cells, which indicates myelin denaturation. Bar indicates 50 μm. This experiment was independently performed at least three times. (DOCX) [file pone.0262892.s002.docx]
